# The effect of limit feeding and delaying implanting until d-64 of the finishing period on growth performance and carcass characteristics of backgrounded beef steers

**DOI:** 10.1093/tas/txag146

**Published:** 2026-09-24

**Authors:** Justin J Delver, Federico Podversich, Ana C B Menezes, Warren C Rusche, Dale A Blasi, Logan R Thompson, K C Olson, John P Ritten, Zachary K F Smith

**Affiliations:** Department of Animal Science, South Dakota State University, Brookings, SD 57007, United States; Department of Animal Science, South Dakota State University, Brookings, SD 57007, United States; Department of Animal Science, South Dakota State University, Brookings, SD 57007, United States; Department of Animal Science, South Dakota State University, Brookings, SD 57007, United States; Department of Animal Sciences and Industry, Kansas State University, Manhattan, KS 66506, United States; Department of Animal Sciences and Industry, Kansas State University, Manhattan, KS 66506, United States; Department of Animal Sciences and Industry, Kansas State University, Manhattan, KS 66506, United States; CSU AgNext, Department of Animal Sciences, Colorado State University, Fort Collins, CO 80523, United States; Department of Animal Science, South Dakota State University, Brookings, SD 57007, United States

**Keywords:** beef, feedlot, intake, steroidal implant

## Abstract

The objective of this study was to determine the effects of feed restriction and implant timing on growth and carcass characteristics of finishing beef steers. Charolais cross steers (*n* = 232; initial shrunk body weight [SBW] = 478 ± 27.4 kg) were allocated into 24 open dirt lot pens (8 steers/pen) and 8 partially covered pens (5 steers/pen). Pens were randomly assigned to one of four treatments in a 2 × 2 factorial arrangement of implant and diet strategy. Implant treatments consisted of administration of 200 mg trenbolone acetate and 20 mg estradiol—17β (Revalor 200; Merck Animal Health, Rahway, NJ) on d-1 (INI) or d-64 (DEL). Diet treatments were ad libitum feeding throughout the feeding period (ADL) or restriction to 92% of ad libitum for 63 d, followed by ad libitum feeding (RES). The RES pens were offered 92% of the intake of the ADL pens within the same implant strategy and block. Data were analyzed using the MIXED procedure in SAS 9.4, with the GLIMMIX procedure used for categorical distributions. Diet, implant strategy, and their interaction were fixed effects, and block was the random effect. There was only one interaction effect (*P* = 0.04) of gain to feed (G: F) from d 64 to 85. From d 1 to 63, dry matter intake (DMI) and average daily gain (ADG) were greater for the INI and ADL groups (*P* < 0.01) while G: F was only greater in the INI group (*P* < 0.01). By d 64, SBW was greater in the INI and ADL groups (*P* < 0.01). During d 64 to 142, DMI did not differ between treatments (*P* ≥ 0.23), however, DMI as a percentage of BW was greater for RES (*P* = 0.05). The DEL group had greater ADG and G: F (*P* < 0.01). Cumulatively, DMI was greater in the INI and ADL groups (*P* ≤ 0.03) but final BW, ADG, and G: F did not differ between the implant or feeding management treatments (*P* ≥ 0.12). Carcass-adjusted final BW (HCW/0.625) and ADG were also similar between treatments (*P* ≥ 0.66), while G: F was only greater in the DEL group (*P* = 0.04). Hot carcass weight and marbling score did not differ (*P* ≥ 0.43). Rib fat was greater in the INI group while ribeye area was greater in the DEL group (*P* ≤ 0.01). Performance calculated energetics did not differ (*P* ≥ 0.10). Yield grade distribution shifted towards more Yield Grades 1 and 2 in the DEL group (*P* = 0.02). Restricting feed intake during the initial 63-d resulted in reduced DMI but similar final SBW and HCW. Delaying implanting had similar results, however rib fat was decreased, and ribeye area was increased.

## Teaser

Limiting feed intake of beef steers for a portion of the finishing period and delaying implant administration can reduce feed intake while maintaining animal performance, with delayed implanting also increasing carcass leanness.

## Introduction

The use of limit feeding in finishing cattle diets improves feed efficiency (Galyean et al. 1999). Limit feeding in the finishing phase can involve a restriction lasting the entire feeding period, or a restriction period followed by ad libitum access to feed. The latter strategy consists of a restriction period, when the feed restriction is being applied, and the realimentation period, when feed restriction is removed and cattle return to ad libitum intake. The realimentation period is important, especially in the finishing period, as this is when cattle can capitalize on compensatory gain. In a previous experiment by our lab group, we observed similar growth performance and improved feed efficiency when cattle were limit fed to 92% of ad libitum intake for 56 or 86 days of a 140-day finishing period (Delver et al. 2024). Although live performance was similar in the restricted and ad libitum groups, the restricted groups had reduced marbling scores, and quality grades were shifted away from Prime grades (Delver et al. 2024). These improvements in feed efficiency are a result of decreased maintenance costs associated with smaller visceral organ mass and a change in carcass composition towards more lean tissue and less fat (Hicks et al. 1990; Murphy and Loerch 1994; Sainz and Bentley 1997). Determining the optimal length of restriction requires balancing sufficient time to elicit this compensatory growth response while allowing adequate realimentation time to recover carcass quality. Because steers programmed to gain 1.13 kg/d for 70 days exhibited greater subsequent compensatory gains than those restricted for 32 or 47 days (Knoblich et al. 1997), as well as the responses from Delver et al. (2024), an intermediate restriction of 63 days was selected for the current study to maximize compensatory gain potential while allowing sufficient realimentation time.

Anabolic growth promoting implants are routinely used in the finishing period to improve growth performance of cattle (Smith and Johnson 2020). However, with those improvements in growth, implanted cattle, especially on an aggressive implant regimen, have decreased marbling at similar days of feed (Johnson et al. 1996). An implant management strategy often employed to help minimize the negative impacts on marbling and carcass quality is delaying implant administration until later in the feeding period (Bruns et al. 2005; Griffin et al. 2009; Barham et al. 2012). Delaying implants also helps better match the payout period of the implant to maximum energy intake (Griffin et al. 2009). The cattle in Delver et al. (2024) were implanted at the initiation of the feed restriction and feeding of the finishing diet. This was necessary to determine the effects of the limit feeding period, however, the effects on carcass characteristics from limit feeding may have been exacerbated by the implant administered at the beginning of the restriction period. In addition to this, much of the payout period of the implant is “wasted” as cattle lack the maximum energy intake required to take full advantage of the technology. By delaying the implant for 63 days to align with the conclusion of the 63-day feed restriction, the payout period of the implant is synchronized with the return to ad libitum intake and the subsequent compensatory growth phase. The objective of this experiment was to determine the effects of a 63-d feed restriction and implant timing during the finishing period on the growth and carcass characteristics of backgrounded beef steers.

## Materials and methods

This study was conducted at the Southeast Research Farm in Beresford, South Dakota, between April 2024 and September 2024. The animal care and handling procedures used in this study were approved by the South Dakota State University Animal Care and Use Committee (2306-052E).

### Treatments

Steers were allotted to 1 of 4 treatments in a 2 × 2 factorial arrangement of implant timing and feeding management and were blocked by pen location in the feedlot and BW. The implant treatments were:

1. Implanted with a 200 mg trenbolone acetate (TBA) and 20 mg estradiol - 17β (E2) implant (Revalor 200, Merck Animal Health, Rahway, NJ) on d 1 (INI)
2. Implanted with a 200 mg TBA and 20 mg E2 implant (Revalor 200, Merck Animal Health,) on d 64 (DEL)

The feeding management treatments were:

1. Offered *ad libitum* access to feed for the entire duration of the study (ADL)
2. Offered 92% of *ad libitum* feed intake from d 1 to d 63, then offered *ad libitum* access to feed for the remainder of the study, d 64 to 142 (RES)

This resulted in four treatment groups: INI-ADL, INI-RES, DEL-ADL, and DEL-RES. The RES restrictions were determined by offering 92% of the ADL groups feed within their respective block and implant treatment. The ADL group for the entire study, and the RES group from d 64 to 142, were managed to have a thin layer of feed remaining in the bunk prior to morning feeding. Weigh backs were collected weekly and that feed was subtracted from that pen’s feed deliveries for the week. Cattle were reweighed and had implants checked 21- or 22-days post-implant administration for implant defects. The INI-ADL had 3 soft inflammations, INI-RES had 1 soft inflammation, DEL-ADL had 1 soft inflammation and 1 abscess, and the DEL-RES had no implant defects. All implants remained correctly in the ear so no reimplants were administered.

### Cattle feeding and management

On March 28, 2024, 242 Charolais cross steers (arrival body weight [BW] = 477 ± 30.5 kg) were transported 489 km from a backgrounding yard near Philip, SD to the Southeast Research Farm in Beresford, SD. Steers were group housed (∼10 steers/pen) and offered their initial diet at 2% of BW on a dry matter (DM) basis. Steers were transitioned to their final diet through 3 step-up diets so that all steers were on their final diets by April 18, 2024. Steers were weighed, administered a visual ear tag, vaccinated against infectious bovine rhinotracheitis virus, parainfluenza3 virus, bovine respiratory syncytial virus; bovine viral diarrhea Types 1a, 1 b, and 2 virus, and *Mannheimia haemolytica* type A1 (Bovishield Gold Oneshot, Zoetis, Parsippany, NJ) and *Clostridium chauvoei, Cl. Novui, Cl. perfringens* Types B, C, and D, and *Histophilus somni* (Ultrabac 7/Somubac, Zoetis), as well as administered a pour-on anthelmintic (Cydectin, Elanco Animal Health, Indianapolis, IN) according to label directions on April 19, 2024. On April 22, 2024 (d 1 of study), steers were reweighed and allocated to treatment pens using the April 19 weights resulting in 232 steers on study with 24 open lot dirt surfaced pens (8 steers/pen; *n* = 192) and 8 partially covered concrete surfaced pens (5 steers/pen; *n* = 40). To produce a similar BW distribution within pens, steers were sorted by their April 19 BW and outliers were removed to keep 232 steers. The heaviest 20 and lightest 20 steers were blocked by BW and assigned to 1 of 4 treatments. These two replicates were housed in the partially covered concrete surfaced pens. The remaining steers were sorted by BW and assigned alternatively to replicates 1 to 6. Steers were then sorted by replicate and BW and pseudo-randomly assigned to 1 of 4 treatments and assigned their open lot dirt surfaced pen. On d 1, steers that were in the INI implant treatment were administered their implant and steers that were in the RES feeding treatment began their feed restriction of 92% of ad libitum intake.

Steers were housed in open lot dirt pens (24 pens; 192 steers; 35 m × 14 m) with 6 m concrete bunks and 3 m concrete aprons or partially covered concrete surfaced pens (8 pens, 40 steers; 5 m × 12 m) with 4.9 m of bunk space. All pens were equipped with heated continuous flow water troughs that were shared between two pens. Individual ingredient samples were collected weekly and analyzed for DM. Weekly ingredient samples were composited by month and analyzed for N (method no. 968.06; AOAC 2016; Rapid Max N Exceed, Elementar, Mt. Laurel, NJ) and ash (method no. 942.05; AOAC 2012). Dry rolled corn was not analyzed for neutral detergent fiber (NDF) or acid detergent fiber (ADF) and assumed an NDF and ADF content of 9% and 3%, respectively (Preston 2017); while sorghum silage and modified distillers plus solubles (MDGS) were analyzed for NDF and ADF according to Goering and Soest (1970) with the addition of sodium sulfite and alpha amylase to control for protein contamination. Tabular ether extract (EE) values were used for all ingredients except MDGS, which were analyzed using an Ankom Fat Extractor (XT10: Ankom Technology, Macedon, NY). Actual diet formulation based upon weekly DM determination and feed batching records with tabular energy values according to Preston (2017) are presented in Table 1. Steers were fed once daily at 0800 with feed mixed in a commercial mixer wagon (6.1 m^3^; Reel Auggie 3120, Kuhn North America, Inc., Brodhead, WI) with a scale resolution of 0.91 kg. Environmental data was retrieved from the South Dakota Mesonet climate recording station located onsite (South Dakota State University 2026). The comprehensive climate index (CCI) was calculated using daily average ambient temperature, wind speed, relative humidity, and solar radiation according to Mader et al. (2010). During the study duration, 73% of the days had missing solar radiation measurements, primarily from June to September. The missing solar radiation values were replaced with average solar radiation values for Beresford from May to September of 2024. Minimum and maximum ambient temperatures were the daily low and high temperatures, respectively. The mean ambient temperature as well as the mean, minimum, and maximum relative humidity, wind speed, and CCI were calculated from daily averages (Table 2).

**Table 1:** Ingredient and nutrient composition of diets fed to backgrounded Charolais cross beef steers.

	Diet 1 (d 1 to 119)	Diet 2 (d 120 to 142)
**Ingredients, % DM**		
**Dry Rolled Corn**	70.5	58.9
**MDGS^1^**	17.5	15.4
**Sorghum Silage**	8.0	–
**Corn Silage**	–	21.7
**Suspended Supplement^2^**	4.0	4.0
**Nutrient Composition, Dry Matter Basis**		
**Dry Matter, % As Fed**	66.7	59.7
**Crude Protein, %**	13.2	12.6
**Neutral Detergent Fiber, %**	17.7	23.3
**Acid Detergent Fiber, %**	9.2	11.0
**Ash, %**	5.1	5.3
**Ether Extract, %**	3.9	3.7
**Net Energy for Maintenance, Mcal/kg**	2.08	2.04
**Net Energy for Gain, Mcal/kg**	1.40	1.37
**Monensin, mg/kg**	32.7	32.8

1 Modified distillers’ grains plus solubles.

2 Suspended supplement contained (DM basis): 27% crude protein, 22.25% non-protein nitrogen, 0.80 Mcal/kg NE for maintenance, 0.54 Mcal/kg NE for gain, 0.09% ether extract, 16.92% Ca, 0.4% *P*, 1.54% K, 0.24% Mg, 7.92% NaCl, 3.31% Na, 0.43% S, 4.62 ppm Co, 250 ppm Cu, 40 ppm I, 50 ppm EDDI, 244.93 ppm Fe, 500 ppm Mn, 4 ppm Se, 2253.85 ppm Zn, 79380 IU/kg vitamin A, 19845 IU/kg vitamin D3, 551 IU/kg vitamin E, 827.7 mg/kg monensin sodium.

**Table 2:** The environmental conditions during the duration of a study (April 22, 2024 to September 10, 2024) evaluating a 2 × 2 factorial treatment design of a hormonal implant administered on d 1 (INI) or d 64 (DEL) and offering feed ad libitum (ADL) or limiting feed to 92% of ad libitum feed intake for 63 d (RES) before being offered ad libitum feed for the remaining 78 d in backgrounded beef steers.

	Mean	SD	Min	Max
**Ambient temperature, °C**	20	4.1	−3	35
**Relative humidity, %**	72	11.8	36	93
**Wind speed, m/s**	4.2	1.75	1.3	9.8
**CCI^1^, °C**	19	5.3	6	30
**CCI^2^ ≥ 30, d**	1	–	–	–
**CCI^3^ ≥ 25, d**	23	–	–	–
**CCI^4^ ≥ 22, d**	47	–	–	–

1 Comprehensive Climate Index calculated from Mader et al. (2010).

2 CCI ≥ 30°C indicates moderate heat stress (Mader et al. 2010).

3 CCI ≥ 25°C indicates mild heat stress (Mader et al. 2010).

4 CCI ≥ 22°C indicates potential for heat stress in fat cattle with sustained CCI and little nighttime cooling (Brown-Brandl et al. 2006; Mader et al. 2010).

### Growth performance

All steers were individually weighed on days -3, 1, 22, 64, 86, and 142. Steers were weighed in a hydraulic squeeze chute mounted on top of load cells (scale readability ± 0.45 kg) prior to morning feeding. All BW measurements were pencil shrunk 4% to account for gastrointestinal fill (SBW). Initial SBW was calculated as the average of the d -3 and 1 BW shrunk 4%. Metabolic body weight (MBW) was calculated for each period as the median shrunk body weight (MeBW) of each feeding period to the power of 0.75. Dry matter intake (DMI) as a percentage of BW and MBW was calculated as the average DMI of the feeding period divided by the median BW and MBW of the feeding period, respectively. Carcass adjusted final BW was calculated as hot carcass weight (HCW)/0.625. A standard reference dressing percent of 62.5% was used to normalize final BW and growth performance independent of gut fill. Average daily gain (ADG) was calculated as the difference between final SBW and initial SBW divided by days on feed (DOF) for each weigh period and feed conversion efficiency (G: F) was calculated from ADG/DMI.

### Carcass characteristics

Steers were fed until visually appraised to have 1.54 cm rib-fat. Steers were weighed off study the morning of d 142 and radio-frequency identification (RFID) tags were applied. Cattle were transported 211 km to a commercial beef abattoir that afternoon to be harvested the following morning (September 11, 2024). Trained individuals entered the slaughter facility, and individual visual identification tags were recorded, RFID tags were recorded via Allflex RS420NFC Series Stick Reader (Allflex USA LLC, Rahway, NJ), and packer identification tags were recorded to ensure individual carcasses could be traced to live steers. Livers were also evaluated for health according to the Elanco Liver Check Service (Elanco U.S., Inc., Greenfield, IN). Following chilling, all carcasses were ribbed for USDA-AMS grading, and quality and yield grade attributes were obtained with camera grading. Dressing percentage was calculated as HCW/Final SBW. Estimated empty body fat (EBF) percentage, empty body weight (EBW), and final BW at 28% EBF (AFBW) were calculated from observed carcass traits (Guiroy et al. 2002). The m-score was calculated for each steer using the following equation:


$$\left[\frac{{(y}_{1i}-{\bar{y}}_{1})}{s_{y1}}]-[\frac{\left(y_{2i}-{\bar{y}}_{2}\right)}{s_{y2}}\right]$$

where $y_{1i}$ is the individual steers marbling score, ${\bar{y}}_{1}$ is the mean marbling score of all the steers, $s_{y1}$ is the standard deviation of all the marbling score for all the steers, $y_{2i}$ is the individual steers rib fat thickness, ${\bar{y}}_{2}$ is the mean rib fat thickness of all the steers, and $s_{y2}$ is the standard deviation of the rib fat thickness for all the steers.

### Efficiency of dietary NE utilization

Observed dietary net energy (NE) and the ratio of observed-to-expected (O: E) dietary NE were assessed for the cumulative feeding period (d 1 to d 142). Carcass-adjusted growth performance was used to calculate performance-based dietary NE to determine efficiency of dietary NE utilization. The performance-based dietary NE was calculated from energy gain (EG; Mcal/d): EG = ADG^1.097^ × 0.0557 W^0.75^; where W is the mean equivalent shrunk body weight (EQSBW; kg; NASEM 2000) calculated from MeBW and AFBW: EQSBW = MeBW × (478/AFBW). Maintenance energy (EM) was calculated as: EM = 0.077 × MBW. Dry matter intake is related to energy requirements and dietary net energy for maintenance (NEm; Mcal/kg) according to the following equation: DMI = EG/(0.877NEm − 0.41), and can be resolved for estimation of dietary NEm by means of the quadratic formula x = (-b±√[b^2^-4ac])/2c, where a = −0.41EM, b = 0.877EM + 0.41DMI + EG, and c = −0.877DMI (Zinn and Shen 1998). Dietary net energy for gain (NEg; Mcal/kg) can be calculated as: NEg = 0.877NEm − 0.41 (Zinn 1987). Observed-to-expected NEm and NEg were a ratio of performance-based dietary NE to tabular dietary NE values (Preston 2017). A ratio of <1 indicates that the calculated NE of the diet based on the observed cattle performance was less than expected based on the tabular NE of the diet. A ratio of >1 indicates that the calculated NE of the diet based on the observed cattle performance was greater than expected based on the tabular NE of the diet. The maintenance coefficient (MQ; Mcal/kg^0.75^) was determined using the following equation: MQ = ([DMI – {EG/Neg}] × Nem)/MBW.

### Statistical analysis

All data were analyzed with the fixed effects of implant treatment, diet treatment, and the implant × diet interaction and the random effect of block. Growth performance, carcass characteristics, and efficiency of dietary NE utilization were analyzed using the MIXED procedure of SAS 9.4 (SAS Inst. Inc., Cary, NC) with pen as the experimental unit. Least square means were generated with the LSMEANS option of PROC MIXED and means were separated and denoted different (*P* ≤ 0.05) or a tendency to be different (0.05 < *P* ≤ 0.10) using the least significant difference test. Quality Grade, Yield Grade, and liver scores were analyzed using the GLIMMIX procedure of SAS 9.4 with pen as the experimental unit using a multinomial distribution. Significance was determined at *P* ≤ 0.05 and tendencies at 0.05 < *P* ≤ 0.10.

## Results

There was only one implant × diet interaction, which was from d 64 to 85 (*P* = 0.04; Table 3) where the DEL-RES group had greater G: F than the other three treatments. There was also a tendency for an interaction from d 64 to 85 for ADG (*P* = 0.06). The interaction effects on growth performance during the restriction, realimentation, and cumulative periods were not different (*P* ≥ 0.17). The interaction effects on carcass characteristics and energetics (*P* ≥ 0.14; Table 4) as well as Yield Grade, Quality Grade, and liver score distribution were not different (*P* ≥ 0.50; Table 5).

**Table 3:** The effects on growth performance during the interim, restriction, realimentation, and cumulative period of backgrounded beef steers in a 2 × 2 factorial treatment design evaluating a hormonal implant administered on d 1 (INI) or d 64 (DEL) and offering feed ad libitum (ADL) or limiting feed to 92% of ad libitum feed intake for 63 d (RES) before being offered ad libitum feed for the remaining 78 d.

	INI		DEL			*P*-value		
	ADL	RES	ADL	RES	SEM^1^	Implant × Diet	Implant	Diet
**d 1 to 21**								
**Initial Shrunk Body Weight^2^, kg**	459	460	459	459	8.5	0.72	0.53	0.42
**Dry Matter Intake, kg/d**	11.64	10.63	11.33	10.42	0.146	0.54	<0.01	<0.01
**Dry Matter Intake^3^, % BW**	2.38	2.18	2.35	2.18	0.022	0.42	0.35	<0.01
**Dry Matter Intake^4^, % MBW**	13.62	12.49	13.40	12.42	0.102	0.45	0.14	<0.01
**Average Daily Gain, kg/d**	3.02	2.63	2.41	1.88	0.139	0.47	<0.01	<0.01
**Gain: Feed**	0.260	0.247	0.213	0.180	0.0133	0.27	<0.01	0.02
**d 22 to 63**								
**d 22 SBW, kg**	522	515	510	499	7.9	0.37	<0.01	<0.01
**Dry Matter Intake, kg/d**	12.49	11.61	11.88	11.12	0.343	0.73	<0.01	<0.01
**Dry Matter Intake^3^, % BW**	2.22	2.09	2.19	2.09	0.046	0.53	0.72	<0.01
**Dry Matter Intake^4^, % MBW**	13.15	12.34	12.86	12.24	0.288	0.58	0.28	<0.01
**Average Daily Gain, kg/d**	1.99	1.98	1.66	1.60	0.079	0.77	<0.01	0.60
**Gain: Feed**	0.159	0.171	0.139	0.144	0.0043	0.42	<0.01	0.07
**d 64 to 85**								
**d 64 SBW, kg**	606	598	579	566	9.6	0.43	<0.01	0.01
**Dry Matter Intake, kg/d**	10.84	10.98	10.51	10.80	0.212	0.67	0.15	0.24
**Dry Matter Intake^3^, % BW**	1.74	1.78	1.76	1.83	0.034	0.56	0.28	0.05
**Dry Matter Intake^4^, % MBW**	10.60	10.82	10.59	10.99	0.193	0.59	0.64	0.08
**Average Daily Gain, kg/d**	1.70	1.70	1.89	2.32	0.128	0.06	<0.01	0.07
**Gain: Feed**	0.156^b^	0.155^b^	0.180^b^	0.215^a^	0.0114	0.04	<0.01	0.06
**d 86 to 142**								
**d 86 SBW, kg**	644	636	621	617	9.6	0.59	<0.01	0.16
**Dry Matter Intake, kg/d**	11.48	11.55	11.24	11.49	0.239	0.58	0.34	0.33
**Dry Matter Intake^3^, % BW**	1.70	1.72	1.69	1.74	0.024	0.52	0.79	0.09
**Dry Matter Intake^4^, % MBW**	10.54	10.66	10.45	10.73	0.158	0.53	0.94	0.13
**Average Daily Gain, kg/d**	1.23	1.37	1.65	1.65	0.080	0.22	<0.01	0.21
**Gain: Feed**	0.108	0.118	0.146	0.143	0.0059	0.18	<0.01	0.38
**d 142 SBW, kg**	713	712	713	709	10.8	0.70	0.76	0.65
**d 1 to 63**								
**Dry Matter Intake, kg/d**	12.21	11.28	11.70	10.89	0.266	0.67	<0.01	<0.01
**Dry Matter Intake^3^, % BW**	2.30	2.13	2.26	2.13	0.036	0.49	0.39	<0.01
**Dry Matter Intake^4^, % MBW**	13.43	12.46	13.13	12.33	0.216	0.54	0.14	<0.01
**Average Daily Gain, kg/d**	2.33	2.19	1.91	1.70	0.079	0.51	<0.01	<0.01
**Gain: Feed**	0.191	0.195	0.163	0.155	0.0053	0.17	<0.01	0.56
**d 64 to 142**								
**Dry Matter Intake, kg/d**	11.31	11.40	11.04	11.30	0.218	0.57	0.23	0.26
**Dry Matter Intake^3^, % BW**	1.72	1.74	1.71	1.78	0.024	0.34	0.51	0.05
**Dry Matter Intake^4^, % MBW**	10.61	10.74	10.51	10.87	0.15	0.39	0.87	0.07
**Average Daily Gain, kg/d**	1.36	1.46	1.71	1.84	0.061	0.81	<0.01	0.05
**Gain: Feed**	0.121	0.128	0.155	0.163	0.0043	0.96	<0.01	0.09
**d 1 to 142**								
**Dry Matter Intake, kg/d**	11.71	11.35	11.34	11.12	0.226	0.55	0.02	0.03
**Dry Matter Intake^3^, % BW**	2.00	1.94	1.94	1.91	0.026	0.39	0.02	0.02
**Dry Matter Intake^4^, % MBW**	11.99	11.62	11.61	11.42	0.163	0.43	0.02	0.02
**Average Daily Gain, kg/d**	1.80	1.79	1.80	1.77	0.051	0.76	0.85	0.54
**Gain: Feed**	0.154	0.158	0.159	0.160	0.0032	0.50	0.12	0.30
**d 1 to 142 Carcass-Adjusted^5^**								
**Average Daily Gain, kg/d**	1.93	1.94	1.95	1.94	0.044	0.66	0.66	0.97
**Gain: Feed**	0.171	0.172	0.173	0.172	0.0039	0.66	0.66	0.97
**d 142 SBW, kg**	733	736	736	736	11.3	0.67	0.71	0.80

ab Means within a row lacking a common superscript differ *P* ≤ 0.05.

1 SEM presented is for the interaction of implant × diet effect.

2 A 4% pencil shrink was applied to all BW measures to account for gastrointestinal tract fill.

3 Dry matter intake as a percentage of median shrunk body weight for the period.

4 Dry matter intake as a percentage of median metabolic body weight for the period.

5 Carcass adjusted final body weight calculated from HCW/0.625.

**Table 4:** The effects on carcass characteristics and energetics of backgrounded beef steers in a 2 × 2 factorial treatment design evaluating a hormonal implant administered on d 1 (INI) or d 64 (DEL) and offering feed ad libitum (ADL) or limiting feed to 92% of ad libitum feed intake for 63 d (RES) before being offered ad libitum feed for the remaining 78 d.

	INI		DEL			*P*-value		
	ADL	RES	ADL	RES	SEM^1^	Implant × Diet	Implant	Diet
**Carcass Characteristics**								
**Hot Carcass Weight, kg**	458	460	460	460	7.1	0.67	0.71	0.80
**Dressing Percent, %**	64.28	64.57	64.56	64.54	0.265	0.44	0.55	0.50
**Ribeye Area, cm^2^**	94.5	97.3	99.1	99.3	1.52	0.30	0.01	0.22
**Rib Fat, cm**	1.58	1.54	1.41	1.41	0.058	0.67	<0.01	0.67
**Marbling^2^**	505	508	527	501	16.3	0.32	0.59	0.43
**Estimated EBF^3^, %**	32.24	31.87	31.28	31.07	0.435	0.81	0.01	0.37
**Estimated EBW^4^, kg**	635	637	638	637	9.3	0.67	0.71	0.80
**AFBW^5^, kg**	645	654	663	666	7.4	0.62	0.02	0.35
**M-Score^6^**	−0.230	−0.122	0.304	0.044	0.1295	0.14	<0.01	0.54
**Performance Calculated Dietary Net Energy**								
**Maintenance, Mcal/kg**	2.06	2.10	2.09	2.12	0.025	0.84	0.24	0.18
**Gain, Mcal/kg**	1.40	1.43	1.43	1.45	0.022	0.84	0.24	0.18
**MQ^7^, Mcal/BW^0.75^**	0.078	0.073	0.074	0.071	0.0029	0.75	0.24	0.17
**Observed: Expected Ratio**								
**Net Energy for Maintenance**	0.99	1.01	1.01	1.02	0.012	0.84	0.24	0.18
**Net Energy for Gain**	1.00	1.02	1.02	1.04	0.016	0.84	0.24	0.18
**Dry Matter Intake**	1.01	0.99	0.99	0.98	0.014	0.77	0.20	0.10

1 SEM presented is for the interaction of implant × diet effect.

2 400 = Small^00^.

3 Estimated percentage empty body fat calculated from Guiroy et al. (2002) = 17.76207 + (4.68142 × fat thickness, cm) + (0.01945 × hot carcass weight, kg) + (0.81855 × QG) – (0.06754 × ribeye area, cm^2^); QG = quality grade (Select = 4, Low Choice = 5, Average Choice = 6, High Choice = 7, Low Prime = 8).

4 Estimated empty body weight was calculated from Guiroy et al. (2002) = (1.316 × hot carcass weight, kg) + 32.29.

5 Final body weight adjusted to 28% empty body fat (Guiroy et al. 2002).

6 M-score of marbling vs rib fat calculated as ([marbling score—mean marbling score]/[marbling score standard deviation]) – ([rib fat– mean rib fat]/[rib fat standard deviation]).

7 Calculated maintenance coefficient: ([{dry matter intake—feed required for gain} × {dietary net energy for maintenance}]/[median shrunk body weight]^0.75^).

**Table 5:** The effects on yield grade, quality grade, and liver score distribution of backgrounded beef steers in a 2 × 2 factorial treatment design evaluating a hormonal implant administered on d 1 (INI) or d 64 (DEL) and offering feed ad libitum (ADL) or limiting feed to 92% of ad libitum feed intake for 63 d (RES) before being offered ad libitum feed for the remaining 78 d.

	INI		DEL		*P*-value		
	ADL	RES	ADL	RES	Implant × Diet	Implant	Diet
**Yield Grade, %**					0.62	0.02	0.30
**1**	2.0	2.9	4.1	4.7			
**2**	16.6	22.4	28.6	31.2			
**3**	50.7	51.7	50.1	48.8			
**4**	24.5	18.7	14.2	12.7			
**5**	6.2	4.3	3.0	2.6			
**Quality Grade, %**					0.50	0.92	0.40
**Select**	13.4	14.0	11.4	15.7			
**Low Choice**	38.3	38.8	35.6	40.5			
**Average Choice**	30.9	30.4	32.7	28.9			
**Upper Choice**	12.2	11.8	14.1	10.6			
**Prime**	5.2	5.0	6.2	4.2			
**Liver Score^1^, %**					0.75	0.12	0.14
**No abscess**	77.2	87.6	87.9	92.2			
**Minor**	9.7	5.7	5.5	3.7			
**Major**	13.1	6.8	6.6	4.2			

1 Evaluated according to the scoring system of Brown and Lawrence (2010): Edible (no abscesses); Minor = A- (1 or 2 small abscesses or inactive scars), A (1 to 2 large abscesses or multiple small abscesses); Major = A+ (multiple large abscesses), A + Adhesion (liver adhered to gastrointestinal tract or diaphragm or both), or A + Open (open liver abscess).

The mean ambient temperature, relative humidity, and wind speed during this study were 20 ± 4.1°C, 72 ± 11.8%, 4.2 ± 1.75 m/s, respectively (Table 2). The minimum and maximum ambient temperature, relative humidity, and wind speeds were -3 and 35°C, 36 and 93%, and 1.3 and 9.8 m/s, respectively. The mean calculated CCI was 19 ± 5.3°C with a minimum of 6°C and a maximum of 30°C. There were 47, 23, and 1 days in this study where the daily CCI was ≥22, 25, and 30°C, respectively.

### Implant timing

#### Growth performance

The interim growth periods correspond to the periods between body weight measurements, which were d 1 to 21, d 22 to 63, d 64 to 85, and d 86 to 142. Initial SBW did not differ between implant groups, but from d 1 to 21, DMI (kg/d), ADG, and G: F were 2.4, 27.0, and 25.3% greater, respectively, for the INI group (*P* < 0.01; Table 3) but DMI as a percentage of BW and MBW did not differ (*P* ≥ 0.14). Day 22 SBW was 2.9% greater for the INI group (*P* < 0.01) and from d 22 to 63 DMI, ADG, and G: F were 4.7, 19.9, and 15.0% greater, respectively, for the INI group (*P* < 0.01), but again, DMI as a percentage of BW and MBW did not differ (*P* ≥ 0.28). Day 64 SBW was 4.9% greater for the INI group (*P* < 0.01). From d 64 to 85, DMI on a kg/d, percentage of BW, and percentage of MBW basis did not differ between implant groups (*P* ≥ 0.15), but ADG and G: F were 21.1 and 23.2% greater, respectively, for the DEL group (*P* < 0.01). Day 86 SBW was 3.3% greater for the INI group (*P* < 0.01) but d 142 SBW did not differ between implant groups (*P* = 0.76). From d 86 to 142, no measures of DMI differed between implant groups (*P* ≥ 0.34) but ADG and G: F were 23.7 and 24.8% greater, respectively, for the DEL group (*P* < 0.01).

From d 1 to 63, cattle in the DEL group were not implanted yet while the INI group was administered an implant on d 1. During this period, DMI (kg/d), ADG, and G: F were greater for the INI group by 4.0, 22.7, and 19.3%, respectively (*P* < 0.01; Table 3). Dry matter intake as a percentage of BW and MBW did not differ (*P* ≥ 0.14).

From d 64 to 142, all steers were exposed to exogenous steroidal hormones, with the INI group implanted on d 1 and the DEL group implanted on d 64. During this period, all measures of DMI did not differ between implant groups (*P* ≥ 0.23; Table 3), but ADG and G: F were greater for the DEL group by 23.2 and 24.7%, respectively (*P* < 0.01).

Cumulatively, the INI group had 2.6, 2.6, and 2.5% greater DMI as a kg/d, percentage of BW, and percentage of MBW, respectively (*P* ≤ 0.02; Table 3), but ADG and G: F did not differ between implant groups (*P* ≥ 0.12). When the cumulative growth responses were analyzed on a carcass-adjusted basis, ADG, G: F, and d 142 SBW did not differ between implant groups (*P* ≥ 0.66).

#### Carcass characteristics

Hot carcass weight, dressing percent, marbling, and EBW did not differ between implant groups (*P* ≥ 0.55; Table 4). Rib fat thickness and EBF were 10.1 and 2.8% greater, respectively, in the INI group (*P* ≤ 0.01) while ribeye area (REA) and AFBW were 3.4 and 2.4% greater in the DEL group (*P* ≤ 0.02). The marbling vs. rib fat m-score was greater in the DEL group than the INI group (*P* < 0.01). Quality Grade and liver score distribution did not differ between implant groups (*P* ≥ 0.12; Table 5) but Yield Grade distribution did differ (*P* = 0.02). The DEL group had greater Yield Grade 1 s and 2 s and less 4 s and 5 s.

#### Performance calculated energetics

Performance calculated NEm, NEg, and MQ did not differ between treatments, nor did the O: E NEm, NEg, and DMI (*P* ≥ 0.20; Table 4).

### Feeding management

#### Growth performance

Initial SBW did not differ between diet groups (*P* = 0.42; Table 3) but from d 1 to 21 DMI (kg/d, % BW, and % MBW), ADG, and G: F were 8.8, 7.9, 8.1, 18.5, and 10.2% greater, respectively, for the ADL group (*P* ≤ 0.02). Day 22 and 64 SBW were 1.8 and 1.9% greater, respectively, for the ADL group (*P* < 0.01), but the d 86 and 142 SBW did not differ (*P* ≥ 0.16). From d 22 to 63, all measures of DMI (kg/d, % BW, and % MBW) were 7.0, 5.1, and 5.7% greater, respectively, for the ADL group (*P* < 0.01) and G: F tended to be 5.2% greater for the ADL group (*P* = 0.07). Average daily gain did not differ between groups from d 22 to 63 (*P* = 0.60). From d 64 to 85 DMI on a kg/d basis did not differ between diet groups (*P* = 0.24) but DMI as a percentage of BW and MBW tended to be 3.4 and 2.9% greater, respectively, in the RES group (*P* ≤ 0.08). During the final interim period from d 86 to 142, DMI on a kg/d and percentage of MBW basis, ADG, and G: F did not differ between the diet groups (*P* ≥ 0.13), but DMI as a percentage of BW tended to be 2.3% greater for the RES group (*P* = 0.09).

Restrictions began on d 1 and ended on d 63 for the RES group, and during this period, all measures of DMI (kg/d, % BW, and % MBW) and ADG were 7.5, 6.8, 6.9, and 8.4% greater, respectively, in the ADL group (*P* < 0.01; Table 3) but G: F did not differ (*P* = 0.56).

From d 64 to 142, both diet treatments were offered ad libitum access to feed. Dry matter intake on a kg/d basis did not differ between diet groups during this period (*P* = 0.26; Table 3) but was 2.3% greater for the RES group as a percentage of BW (*P* = 0.05) and tended to be 2.3% greater as a percentage of MBW (*P* = 0.07). Average daily gain and G: F tended to be 6.9 and 4.9% greater, respectively, for the RES group as well (*P* ≤ 0.09).

When analyzed cumulatively, DMI on a kg/d, percentage of BW, and percentage of MBW were greater for the ADL group by 2.5, 2.6, and 2.4%, respectively (*P* = 0.03; Table 3) but ADG and G: F did not differ (*P* ≥ 0.30). When the cumulative results were carcass adjusted, ADG, G: F and d 142 SBW did not differ by diet treatment (*P* ≥ 0.80).

#### Carcass characteristics

None of the measured carcass characteristics differed between the ADL and RES diet treatments (*P* ≥ 0.22; Table 4). Yield grade, quality grade, and liver scores also did not differ between diet treatments (*P* ≥ 0.50; Table 5).

#### Performance calculated energetics

The performance calculated NEm, NEg, and MQ did not differ between the dietary treatments (*P* ≥ 0.17; Table 4). The O: E NEm, NEg, and DMI also did not differ between diet treatments (*P* ≥ 0.10).

## Discussion

There was only one significant interaction and one tendency for an interaction in this experiment, and both occurred in the interim period from d 64 to 85. During this period, cattle in the RES group had been returned to ad libitum feed and cattle in the DEL group had been implanted on d 64. The realimentation period and delayed implant had a positive associative effect where the DEL-RES group had greater G: F and tended to have greater ADG than the other treatments. When cattle were offered ad libitum feed in the INI group, both diet groups performed similar, but the DEL-ADL group had greater G: F than the INI-ADL and the DEL-RES had greater G: F than the DEL-ADL. Dry matter intake did not differ during this interim period, even when adjusted for BW or MBW, indicating that these performance differences were driven by physiological differences rather than intake. During early realimentation, cattle have lower maintenance requirements due to smaller visceral organ and gastrointestinal tract mass (Sainz and Bentley 1997). By introducing a TBA and E2 implant at the start of the realimentation period, the somatotropic axis is upregulated, resulting in an increase in circulating somatotropin and insulin-like growth factor-1 (IGF-1) while increasing tissue responsiveness to these hormones (Smith and Johnson 2020). The reduction in maintenance requirements along with the increase in protein deposition driven by the implant may have had a synergistic effect of improving ADG and G: F greater than either the delayed implant or restricted feeding could on its own.

### Implant timing

Steers that were implanted on d 1 outperformed the DEL group for the first 63 d, however, once the DEL group was implanted on d 64, they outperformed the INI group for the final 79 d. Over the entire feeding period, this resulted in no difference in gain, efficiency, or final weight but DEL consumed less DM cumulatively. The carcass characteristics did differ however, with the DEL group having heavier muscled and leaner carcasses but no difference in marbling or quality.

During the initial 63 d, the live performance responses are primarily driven by improved efficiency rather than increased intake. Although the INI group did have greater DMI per day than the DEL group, as a percentage of BW and MBW, they did not differ, which suggests that the intake difference is simply a factor of increased BW in the INI group. The high potency implant (200 mg TBA and 20 mg E2) that the cattle were administered helps increase protein growth while also decreasing protein degradation or turnover (Smith and Johnson 2020). This explains the improved feed efficiency and ADG during the initial 63 d and is an anticipated growth response to hormonal implants (Preston 1999; Guiroy et al. 2002; Smith and Johnson 2020).

On d 64 the DEL group was administered their implants, and we observed comparable growth responses in the final 79 d that the INI group had during the initial 63 d. After the DEL cattle had been implanted, growth performance increased and they outperformed the INI cattle for the final 79 d. Once again, the growth response was not driven by greater intake but by greater feed efficiency. Especially in the case of the DEL cattle, it is likely that the improvement in efficiency is largely driven by decreased protein turnover, increased protein deposition, and decreased fat deposition (Bruns et al. 2005; Smith and Johnson 2020). This is further evidenced by the increased REA and decreased rib fat and EBF in the DEL cattle. Bruns et al. (2005) also delayed implant administration until d 56 in a 150-d finishing period, but they did not observe a difference in carcass characteristic. The cattle were slaughtered at an earlier maturity, 95 and 110% of AFBW in the initial implant treatment groups in Bruns et al. (2005) and the present study, respectively, which may explain why we observed different outcomes. They also measured the fractional accretion rate of protein as 0.53 and 0.41% prior to the delayed implant and 0.26 and 0.32% after the delayed implant in the early and delayed implanted cattle, respectively, which resulted in no cumulative difference (Bruns et al. 2005). It is possible that the DEL cattle had increased fractional protein accretion at a time when they had greater whole-body protein resulting in the increased REA.

Despite the increased REA and decreased rib fat, the DEL cattle did not have decreased marbling or reduction in Quality Grade. An m-score test comparing marbling and rib fat was conducted to determine whether marbling deposition was occurring concomitantly with subcutaneous fat. A positive m-score indicates that marbling is increasing at a faster rate than rib fat relative to the entire group of cattle. The DEL cattle had a greater m-score than the INI cattle indicating a greater proportion of intramuscular fat relative to subcutaneous fat, despite similar marbling scores. In combination with the increased AFBW and decreased EBF and subcutaneous fat, these compositional differences suggest the potential for DEL cattle to achieve greater marbling scores relative to backfat thickness if fed to greater carcass weights.

The quality grade of the cattle did not differ, but the DEL group had a shift in Yield Grades away from 4 and 5 towards more 1 and 2. This is anticipated based on the similar marbling scores but greater REA and decreased rib fat in the DEL cattle. Generally, delaying implants has little effect on yield grade (Bartle et al. 1992; Scaglia et al. 2004; Griffin et al. 2009; Barham et al. 2012; Kayser et al. 2022), but in Bruns et al. (2005) they observed decreased yield grades with delayed implanting.

One of the primary limitations of this study is that the payout period of the uncoated implant used is ∼120 d, meaning circulating concentrations of TBA and E2 may have declined below the threshold to elicit a growth response. This study is also limited in our ability to identify the mechanisms behind the changes in rib fat deposition relative to marbling. The effect of delaying implant administration on marbling is variable, and it is important to note that EBF % at finishing was ∼31.5% and these steers were started on study at 477 kg. The effects of delaying implants are likely dependent on arrival BW as well as DOF.

### Diet management

During the restriction period from d 1 to 63, the RES group ate 7.5% less than the ADL group which is close to the 8% targeted restriction. During this period, ADG was also reduced by 8.4%, with no difference in feed efficiency, which suggests that the reduction in ADG is strictly a DMI response. Within the restriction period, the greatest growth responses occurred within the first 21 days. Average daily gain was decreased by 18.5% during the initial 21 days and G: F was reduced by 10.2%. From d 22 to 63, the RES cattle had some compensatory growth as their ADG was not different from the ADL group and G: F was 5.2% greater than the ADL group. A strong negative response initially to feed restriction on growth performance has been observed in previous studies by Hicks et al. (1990) and Delver et al. (2024).

Cattle in the RES group demonstrated most of their compensatory growth during the first 22 days of the realimentation period. From d 64 to 142, ADG and G: F tended to be greater by 6.9 and 4.9% in the RES group, respectively. Most of this response came from d 64 to 85, with ADG and G: F increased by 11.0 and 9.6%, respectively in the RES group. During this time, DMI intake did not differ on a kg/d basis but tended to be greater per unit of BW and MBW. This is important as maintenance requirements are based on MBW, so an increase in intake relative to BW means more energy is available for growth (Lofgreen and Garrett 1968). This is likely compounded by the decreased maintenance requirements of cattle after a period of feed restriction (Murphy and Loerch 1994; Sainz and Bentley 1997).

Cumulatively, the restricted cattle ate about 2.5% less than the ad libitum fed cattle, but did not differ in feed efficiency, ADG, or final BW. This is similar to Delver et al. (2024), where after either a 56- or 86-day restriction of 8%, the restricted cattle ate less but did not differ in final BW or ADG, resulting in improved feed efficiency. This study differed from Delver et al. (2024) in that none of the carcass characteristics measured differed in this study, while the restricted groups in Delver et al. (2024) had less rib fat and marbling scores, resulting in less Prime carcasses as well as a greater AFBW. The class of cattle in these two studies were different, with the ad libitum fed group having 20.9% and 5.1% Prime in Delver et al. (2024) and this study, respectively. The realimentation period was also greater in this study, 79 d in this study and 34 and 64 for the two restricted groups in Delver et al. (2024). The longer realimentation period in this experiment may have been sufficiently long to recover from the effects of the restricted feeding period, as has been observed in previous studies (Loerch and Fluharty 1998). It is also important to note that cattle in these two experiments were fed at different times of the year, with this study occurring over the summer where major heat events occurred. Mild heat stress is classified as a CCI of 25 to 30°C and moderate heat stress at 30 to 35°C (Mader et al. 2010). Cattle in this study experienced 1 day of moderate heat stress, 22 days of mild heat stress, and 23 days of a CCI from 22 to 25°C. Consecutive days of mild heat stress can exacerbate the effects of the heat stress and fatter cattle may have a lower heat stress threshold or experience more severe heat stress (Brown-Brandl et al. 2006; Mader et al. 2010). Most of the heat events occurred over consecutive days, with five heat events occurring with a mean daily CCI of ≥20°C lasting at least eight consecutive days. Although, due to missing solar radiation data, the average solar radiation data of the available data was extrapolated to the missing days, which may result in uncertainty in the actual CCI values. This is a limitation of our available CCI values. Limit feeding has been investigated as a strategy to decrease heat stress in beef cattle (Holt et al. 2000).

The performance calculated energetics did not differ between our diet management groups. This also suggests that the realimentation period was long enough to dilute out the effects of the restricted period. The growth and feed efficiency responses in the first 22 d of the realimentation period suggest that maintenance energy requirements were reduced by the restricted period, but the cumulative response does not detect that effect. In Delver et al. (2024), the performance calculated energetics indicated that the restricted groups had lower maintenance energy requirements than the ad libitum fed group, even cumulatively.

In combination with Delver et al. (2024), this study helps resolve questions posed in Delver et al. (2024), that increasing the realimentation period can result in the restricted fed cattle reaching similar final BW and carcass composition. However, it is still partially unclear whether the differences in the outcomes of these two studies are due entirely to a longer realimentation period or if cattle and environmental factors also played a significant role.

## Conclusion

The administration of the hormonal implant resulted in improved gain and feed efficiency for the INI group in the first 63 days and in the DEL group in the final 79 days of feeding. Over the entire feeding period both groups had similar performance, but the DEL group ate less. The greater improvements in gain and feed efficiency at the end of the feeding period in the DEL group translated into carcasses with less fat and greater REA, but interestingly no difference in marbling score. This resulted in no difference in the distribution of quality grades but a shift in Yield Grades towards more 1 and 2. This supports delaying implant administration in backgrounded cattle on feed for ∼140 days, but more research is required with different arrival weights and DOF.

The restricted fed cattle performed as expected during the restriction period with decreased intake and growth, but no difference in feed efficiency. The greatest compensatory gain came during the first 22 d of the realimentation period, with no difference in growth responses during the final 57 days of the realimentation period. Interestingly, final BW, cumulative ADG, and carcass characteristics did not differ between diet treatments. It was likely a result of a sufficiently long realimentation period that allowed the RES cattle to catch up to the ADL group. Despite the lack of difference in growth and efficiency, the RES group ate less than the ADL group over the entire feeding period. This suggests that restricted feeding of cattle for a portion of the finishing period, followed by a 79-day realimentation period results in similar live and carcass outcomes with a reduction in feed intake.

## Data Availability

Data can be made available upon reasonable request to ZKFS.

## List of abbreviations

ADF: acid detergent fiber
ADG: average daily gain
BW: body weight
CCI: comprehensive climate index
EG: daily energy gain
DOF: days on feed
DM: dry matter
DMI: dry matter intake
EBF: empty body fat
EBW: empty body weight
EQSBW: equivalent shrunk body weight
AFBW: estimated body weight at 28% empty body fat
E2: estradiol - 17β
EE: ether extract
EM: maintenance energy required
EDDI: ethylenediamine dihydroiodide
G: F: gain to feed ratio
HCW: hot carcass weight
IGF-1: insulin-like growth factor-1
MeBW: median feeding shrunk body weight
MBW: metabolic body weight
MQ: maintenance coefficient
MDGS: modified distillers plus solubles
NE: net energy
NEg: net energy for gain
NEm: net energy for maintenance
NDF: neutral detergent fiber
O: E: observed to expected
RFID: radio-frequency identification
REA: ribeye area
SBW: shrunk body weight
TBA: trenbolone acetate
